# Theoretical analysis of the evolution of immune memory

**DOI:** 10.1186/1471-2148-10-380

**Published:** 2010-12-08

**Authors:** Frederik Graw, Carsten Magnus, Roland R Regoes

**Affiliations:** 1 Integrative Biology, ETH Zurich, Switzerland

## Abstract

**Background:**

The ability of an immune system to remember pathogens improves the chance of the host to survive a second exposure to the same pathogen. This immunological memory has evolved in response to the pathogen environment of the hosts. In vertebrates, the memory of previous infection is physiologically accomplished by the development of memory T and B cells. Many questions concerning the generation and maintenance of immunological memory are still debated. Is there a limit to how many memory cells a host can generate and maintain? If there is a limit, how should new cells be incorporated into a filled memory compartment? And how many different pathogens should the immune system remember?

**Results:**

In this study, we examine how memory traits evolve as a response to different pathogen environments using an individual-based model. We find that even without a cost related to the maintenance of a memory pool, the positive effect of bigger memory pool sizes saturates. The optimal diversity of a limited memory pool is determined by the probability of re-infection, rather than by the prevalence of a pathogen in the environment, or the frequency of exposure.

**Conclusions:**

Relating immune memory traits to the pathogen environment of the hosts, our population biological framework sheds light on the evolutionary determinants of immune memory.

## Background

During their whole life individuals are exposed to many different pathogens, which can cause diseases and sometimes death. Each new pathogen poses a new challenge for the immune system and its different agents. However, vertebrates have evolved immunological memory - a strategy based on remembering previously encountered pathogens that protects against re-infection. This phenomenon was already recognized by the ancient Greeks: The historian Thucydides noted during an outbreak of plague in Athens that those who recovered from the disease were never attacked twice [1]. The establishment of memory is perhaps "the most important consequence of the adaptive immune system" [2]. Immunological memory is divided into cell and antibody mediated branches. The main players of these branches are T and B cells, respectively. Memory cell populations are maintained for many years sometimes leading to life-long immunity [3-5]. Vaccination stimulates the production of immune cells and is one of the most important interventions of disease management and public health.

Many studies have investigated the molecular and mechanistic aspects of how immunological memory is generated and maintained [6-11]. However, beyond the proximal explanations of this phenomenon there is an increasing interest in the evolutionary conditions that favor immunological memory (broadly understood as the memory of previous pathogen infections) [12-21]. In which situations does it make sense to invest in the generation and maintenance of immunological memory, and should all previous pathogen infections be remembered? There is an obvious benefit to memorize all the different pathogens previously encountered: in case of a re-challenge with the same pathogen individuals have a fitness advantage. But this advantage comes with a cost as the maintenance of the memory cells requires resources. In addition, there is only limited physical space in the body to house all the different cells, e.g. the size of the spleen and lymph nodes imposes an upper limit to the number of memory cells.

Several experiments provide evidence that the memory pool of CD8^+ ^T cells has a size constraint that is regulated by homeostasis [22-24]. A similar homeostatic constraint is observed for B-cell responses [25-27]. However, Vezys et al. [28] showed that the memory CD8^+ ^T cell compartment of mice sequentially infected with different viral pathogens grows in terms of absolute cell numbers. It is still debated, how much memory the immune system is able to store [29,30].

If the size of the memory pool is limited, the question arises how the space for new cells is made available [31]. The preferential loss of old memory cells, also known as attrition, has already been observed experimentally [32-36], although the precise mechanism is still unknown. Are antigen-specific cells that encountered the antigen a long time ago lost first or does the immune system sacrifice pre-existing memory cells at random? Moreover, how many antigen-specific cells should be generated per pathogen encounter to establish a strong protective immune response? The size of the entire memory pool and the characteristics of storing new memory determine the diversity of the individual's memory compartment.

As the characteristics of immunological memory can be interpreted as the result of evolutionary processes, it is legitimate to ask what kind of pathogen environments would favor the evolution of one type of memory or the other. In this paper, we use an individual-based population model to determine how different pathogen environments affect the optimal strategy of generating and storing immunological memory. Our analysis identifies key parameters of the pathogen environment that shape the diversity of the memory pool.

## Methods

In this study, we characterize an individual's immune memory with three traits: (i) The maximal number of memory cells an individual can store (*memory size*), (ii) the number of memory cells which are generated after clearing an infection (*replacement size*, note that when an individual has encountered only a few pathogens, the memory pool is not completely filled. The newly created memory cells are integrated into the memory pool without replacing old cells, but one can think of replacing empty space.) and (iii) the way old memory cells are replaced by recently created memory cells, if the size of the memory pool is constrained (*replacement type*). Here the term memory cell is not used in the strict meaning of one single cell but as an abstract unit of memory responses, such as different cells specific for different epitopes of the same pathogen or different types of memory cells. The defined traits (i) - (iii) are assumed to be inherited by an individual to its offspring.

We develop an individual-based model to identify the optimal strategies in terms of these memory genotypes. A strategy is defined as a combination of the three traits listed above. We successively expose a population of individuals to pathogens and follow the evolution over several generations.

### Pathogen environments

Each pathogen environment consists of a pool of *n_p _*pathogens, each of them characterized by two parameters. For an overview of the parameters used see Table 1. The *occurrence probability p*_occ,*j*_describes the probability that an individual living in this environment is exposed to pathogen *j*. The *basic survival probability p*_surv,*j *_denotes the probability that an individual without any memory against pathogen *j *survives an infection by this pathogen. In the context of epidemiology the terms occurrence probability and basic survival probability are also referred to as prevalence and virulence of the pathogen, respectively.

**Table 1:** Parameter values

Parameter	Value	Biological Relevance
**pathogen** **pool**		

*n_p_*	pathogen pool size, 50	This value is small in comparison to findings in [44,45] (1399-1415 human pathogens) but large in comparison to other theoretical studies, e.g. [18].
*p*_occ,*j*_	occurrence probability of pathogen *j*	depends on the pathogen environment
*p*_surv,*j*_	basic survival probability for pathogen *j*	depends on the pathogen environment

*λ*	reproduction probability, 0.2	probability that one individual reproduces per year; an individual living until age 40 produces on average 5 offspring.

*p*_inf_	probability of infection, 0.1 and 0.5	not experimentally determined yet; might vary between pathogens and depend on the route of trans mission

**individuals**		

*n*_mp,*i*_	memory pool size of individual *i*, maximally 50	the maximal value of 50 memory units guarantees that an individual could theoretically generate one memory unit specific for each pathogen
*n*_rep, *i*_	number of newly generated memory cells of individual *i*	maximally 50
*ζ*	probability to survive until the reproductive age; 0.5 and 0.75	childhood mortality differs from country to country; high in non-developed countries and lower in developed countries [59]
*τ*_*i*_	type of replacement of individual *i*	one of the traits studied; 'random' correspond to homeostasis model [37], 'age-dependent' correspond to attrition model [34,35]

*m_i_*(*j*)	number of memory cells of individual *i *against pathogen *j*	
Θ_*k*_(*t*)	number of individuals of type *k *at time *t*	
*w*	fitness difference	

	number of pathogen exposures per year, 3	arbitrarily defined
	survival curve, concave down- ward	The relation between the number of memory cells and the level of protection has not yet been determined quantitatively; our definition guarantees a fast increase with few memory cells, but also the possibility of leaky protection (see also Discussion).

**Figure 1:**
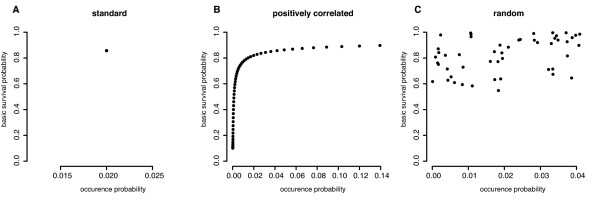
**Pathogen environments**. Pathogen environments are distinguished by the relationship between the occurrence probability of a pathogen in the environment and the probability of an individual without memory to survive an infection with this pathogen (basic survival probability). A standard (**A**), positively correlated (**B**) and random pathogen environment (**C**) are considered. For all environments, the basic survival probability is calculated such that individuals survive the non-reproductive phase with a probability of 50%.

We define three different pathogen environments. In the *standard environment*, each pathogen has the same occurrence probability *p*_occ,*j *_= 1/*n_p _*and a constant basic survival probability *p*_surv,*j *_(Figure 1A). The *positively correlated environment *allows for a positive correlation between the occurrence probability and the basic survival probability. This implies that an individual has a higher survival probability against pathogens, which are more likely to occur than against those, that occur with a lower probability (Figure 1B). In the *random environment*, there is no link between the occurrence probability and the basic survival probability. Both parameters are sampled independently from a uniform distribution (Figure 1C). This random environment is created once and kept fixed for all simulation runs.

For this study we fix the pathogen pool size at *n_p _*= 50. All the three environments are standardized for better comparison (cf. section Simulations).

### Individuals

We keep track of different individuals, which are characterized by three traits: their memory pool size *n*_mp,*i*_, their replacement size *n*_rep,*i *_and their replacement type *τ*_*i*_. The memory pool size of individual *i*, *n*_mp,*i*_, is the number of memory units that individual *i *can store maximally. The maximal memory pool size of a population is the biggest value of all individual memory pool sizes, max*_i_*{*n*_mp,*i*_}. Each individual has a maturation phase which lasts 15 time steps (e.g. corresponding to 15 years). In this phase individuals cannot reproduce. After this phase individuals reproduce within each time step with the *reproduction probability λ*. A newborn individual inherits the memory traits from its parent. Note that only the traits *n*_mp,*i*_, *n*_rep,*i*_, *τ*_*i *_are inherited and not the content of the memory pool itself. The reproduction phase lasts then until age 40 after which the individual is removed from the population, as it does not contribute to the spread of its traits in the population anymore.

If individual *i *is infected with pathogen *j *and has no memory cells against this pathogen, it survives the infection with the probability *p*_surv,*j *_and in this case, it stores *n*_rep,*i *_memory cells of type *j*. However, if individual *i *has already *m_i_*(*j*) memory cells specific for pathogen *j*, the survival probability *S_i_*(*j*) increases according to

(1) $$S_{i}\left(j\right)=p_{\text{surv},j}+\left(1-p_{\text{surv},j}\right)\frac{m_{i}(j)}{\max_{i}\{n_{\text{mp},i}\}}\exp\left(1-\frac{m_{i}(j)}{\max_{i}\{n_{\text{mp},i}\}}\right)$$

**Figure 2:**
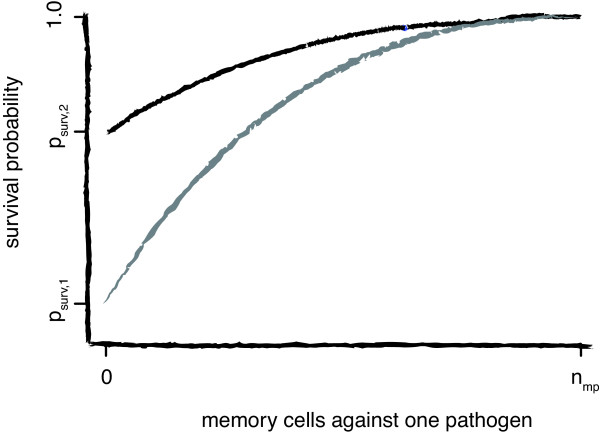
**Effect of memory on the survival probabilities**. Effect of the amount of memory on the probability of an individual to survive a re-exposure with the same pathogen. The probability to survive a pathogen with a low basic survival probability, *p_surv _*(grey) increases faster with an increasing memory response than the survival probability of a pathogen with a higher *p_surv _*(black). Independent of the basic survival probability, a memory pool with maximal pool size occupied by memory cells specific for one pathogen guarantees survival of an infection with this pathogen.

This definition guarantees that an individual with maximal memory pool size that is fully occupied by memory cells specific for one pathogen (*m_i_*(*j*) = max*_i_*{*n*_mp,*i*_}) is completely protected against this pathogen (*S_i _*(*j*) = 1). Note that Equation 1 reduces to the basic survival probability *p*_surv,*j *_if *m_i_*(*j*) = 0 see Figure 2).

In infected individuals that survive infection, the pathogen is cleared and the memory pool is filled with memory cells specific for the encountered pathogens. After some infections, the maximal number of memory cells *n*_mp,*i *_will be reached and old cells have to be erased to make space for the latest memory cells. The replacement type *τ*_*i *_specifies the rule of replacement. In this study, we consider two types of replacement: The *age*-*dependent *replacement type, in which the oldest memory cells are deleted first and the *random *replacement type in which newly created memory cells replace cells of the old memory pool at random [37]. When analyzing one of the memory traits, we keep the other two constant. We assume that there is no fitness cost associated with varying replacement sizes and replacement types. Hence, while testing the replacement size or the replacement type it is not necessary to introduce a fitness cost for the memory pool size. However, maintaining a larger memory pool can come at a cost. Therefore, we analyze the optimal memory pool size first in a cost-free scenario and second in a scenario where a bigger memory pool size is associated with a decrease in the reproductive success.

### Simulations

To determine optimal memory traits, we perform a series of competition experiments between different types of individuals under a constant pathogen environment. The starting population consists of 10000 individuals, which is also the carrying capacity of the population. The initial population in our competition simulations consists of a 1:1 ratio of individuals with the two memory types to be competed with age 0. We chose an initial 1:1 ratio, rather than the more common approach of probing if a rare type can invade the system occupied by the other type, for two reasons: (i) The selection pressure exerted by the pathogen environment does not depend on the frequencies of the different individual types. Therefore, we expect our simulation regime to be equivalent to the common approach. (ii) All the events are of stochastic nature. If one began with a small frequency of one type, the stochastic effects could disguise the selective forces. The selective pressure is highest at a 1:1 ratio.

In the simulations, the time and age is measured in discrete time steps. Within each time step, three pathogens are chosen successively from the pathogen pool in accordance with their occurrence probabilities. Each individual in the population is then exposed to the chosen pathogens, and is infected with a constant probability of infection *p*_inf _. This procedure does not directly account for the epidemiological feedback of the pathogen environment on itself. We adopted this scheme as a compromise between modeling epidemiological feedbacks explicitly, and completely ignoring the accumulation of cases observed in epidemics. The procedure we adopted ensures that many individuals in the population will be infected with the chosen pathogens, and those who do not die develop memory against these pathogens. Furthermore, we assume that all infections lead to an immune response, and do not distinguish between symptomatic and asymptomatic infections.

If an individual *i *is infected with pathogen *j*, it survives with the survival probability *S_i_*(*j*). In this case, the individual generates *n*_rep,*i *_new memory cells specific for pathogen *j*. These memory cells are integrated into the memory pool according to the type of replacement *τ*_*i*_.

After one time step, i.e. three pathogen exposures, surviving individuals that are in the reproductive age reproduce with the probability *λ *= 0.2 and inherit their memory traits to the offspring. The numbers of surviving and newborn individuals are added and if this number exceeds the carrying capacity, we sample 10000 individuals out of the pool of surviving and new born individuals. In the next time step, this routine is repeated with the new population.

The standard, positively correlated and random pathogen environments are standardized for better comparison. The basic survival probabilities and the occurrence probabilities are defined such that an individual survives the non-reproductive phase with a probability of 0.5 given an infection probability per pathogen encounter *p*_inf _= 0.1.

Each simulation is followed over 20000 time steps in total. With this regime, one simulation follows the life-span of roughly 13.6 Mio. individuals and guarantees that an evolutionary steady state is reached. At each time step *t*, the population sizes of the different types of individuals, Θ_*k*_(*t*), *k *= 1, 2, are recorded. To compare the performance of the different types of individuals we estimate the relative fitness between the types, *w*. We fitted the model

(2) $$\log\left(\frac{\Theta_{1}(t)}{\Theta_{2}(t)}\right)=t\log w$$

to our simulated data using a least square algorithm. This is based on the selection model formulated in [38].

Additionally we check for the ratio of the two types at the end of each simulation run, ${\frac{\Theta_{1}(t)}{\Theta_{2}(t)}|}_{t=20000}$. The models and simulations are encoded in the C programming language and loaded into the R language for statistical computing as a shared library. Data analysis was performed with R [39]. The programs are available from the authors upon request.

## Results

### Memory Characteristics

In a first series of simulation experiments we study the fitness of a population characterized by its (i) memory pool size, (ii) replacement size and (iii) replacement type when exposed to pathogen environments that differ in the correlation between the occurrence probabilities of the pathogens and their basic survival probabilities. We consider three different pathogen environments each containing 50 pathogens, namely the *standard, positively correlated *and *random *environment. To allow for comparison, the pathogen environments are calibrated such that individuals survive until the reproductive age with a probability of *ζ *= 0.5. For the following experiments, the probability that an individual is infected with a pathogen upon exposure is defined as *p*_inf _= 0.1. To determine the optimal trait within one memory characteristic we perform competition experiments between populations of individuals differing in this trait while the other two characteristics are kept constant.

#### Memory pool size

**Figure 3:**
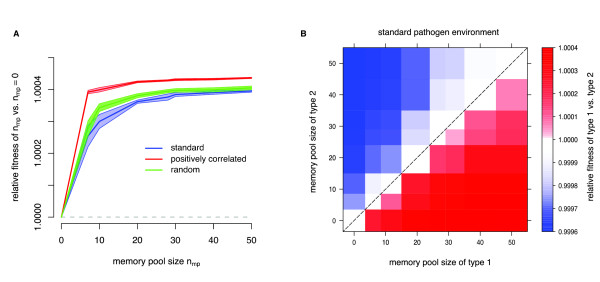
**Optimal memory pool size without cost**. Effect of memory pool size without a cost: **A **Relative fitness as a function of the memory pool size *n*_mp _compared to individuals with no ability to store memory, *n*_mp _= 0. The mean (solid line) as well as the 95% confidence intervals (shaded area) over 15 simulation runs are shown for each pathogen environment. The replacement size was fixed to *n*_rep _= 7. **B **Competition experiments between individuals with different memory pool sizes for the standard pathogen environment, replacement size *n*_rep _= 7 and *age*-*dependent *replacement type. The matrix shows the average fitness value *w *over 15 independent simulation runs. A red rectangle represents a fitness advantage for individuals of type 1, whereas a blue rectangle corresponds to a disadvantage.

To study the effect of various memory pool sizes, we kept the replacement size and the replacement type constant (*n*_rep _= 7, age-dependent). First we assumed that the generation and storage of memory cells is not associated with a fitness cost. In this scenario, we find that it is always beneficial to evolve immune memory. However, while theoretically exposed to 50 different pathogens, the additional advantage of storing more than 20 memory units is only minor as the relative fitness saturates for increasing memory pool sizes (Figure 3A). This saturating effect is observed for all three different pathogen environments. We also tested the optimal memory pool size without cost for different infection scenarios. We varied the probability to survive until the reproductive age, *ζ*, and the probability of being infected upon exposure, *p*_inf_. The saturating effect is also observed under these conditions (see Additional file 1 Figure S4).

**Figure 4:**
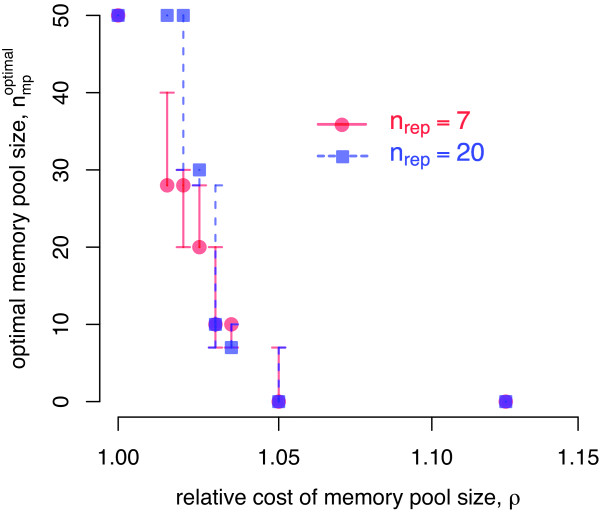
**Optimal memory pool size with cost**. Optimal memory pool size *n*_mp _as a function of the relative cost *ρ*. For each value of *ρ*, competition experiments between individuals differing only in their memory pool size, *n*_mp_, were performed and the relative fitness *w *was determined based on Eq.2. For each combination of (*n*_mp,1_, *n*_mp,2_) with *n*_mp,_. ∈ {0, 7, 10, 20, 28, 30, 40, 50}, we performed 15 independent simulation runs. The figure is based on the following bootstrap routine: For each combination (*n*_mp,1_, *n*_mp,2_) considered, one of the 15 simulation runs was chosen at random and the optimal memory pool size was determined based on this constructed fitness matrix. We performed 10000 replicates per value of *ρ*. The median (dot), as well as the 10% and 90% percentiles are shown. Results are shown for replacement sizes of *n*_rep _= 7 (red) and *n*_rep _= 20 (blue) given the standard pathogen environment.

Arguably, the generation and maintenance of a pool of memory cells is associated with a cost. To implement this possibility, we linked the memory pool size with the reproductive success of an individual via a relative cost, *ρ*. *ρ *= 1 represents a scenario without any cost and *ρ *= 2 corresponds to a situation in which an individual with no memory has a reproduction probability *λ *that is twice as high as that of an individual with the maximal memory pool size of *n*_mp _= 50 (see Additional file 1 for a detailed description). In Figure 4 we show the optimal memory pool size as a function of the relative cost *ρ *for the standard pathogen environment and two different replacement sizes *n*_rep_. The optimal memory pool size decreases with increasing costs. Higher values of *n*_rep _are thereby associated with a wider optimality range of high memory pool sizes and a smaller cost-range for intermediate memory pool sizes.

#### Replacement size

**Figure 5:**
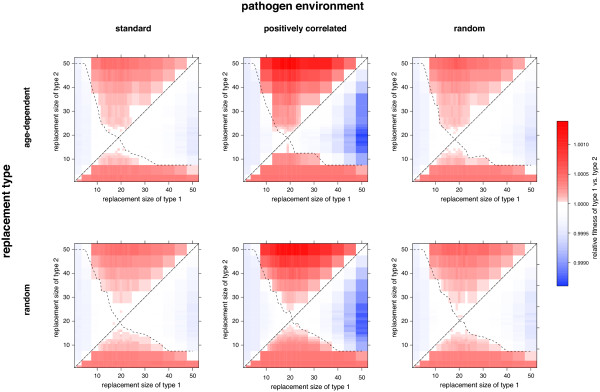
**Optimal replacement size**. Plots of the relative fitnesses in the competition experiments for the three pathogen environments and the two replacement types. The optimal replacement size ranges from 18 to 21. The dashed lines separate the areas, in which one type of individuals performs better than the other. The crossing point of those lines indicates the optimal replacement size.

To determine the optimal number of memory units that an individual should generate and maintain after the survival of an infection, we assumed that each individual holds a maximal memory pool size of *n*_mp _= 50. Hence, for each pathogen one memory unit could be stored theoretically. Similar to our analysis of the memory pool size, we calculated the mean relative fitness over 15 independent simulations of competition experiments between individuals. Here, individuals only differ in the replacement size *n*_rep_. Independent of the three different pathogen environments and of the two replacement types, we obtain an optimal strategy of $n_{\text{rep}}^{\text{optimal}}\approx 19$ (Figure 5) corresponding to approximately 40% of the entire memory pool.

Although the same pattern can be observed in all pathogen environments, the actual values of the relative fitness *w *differ between environments. The positively correlated pathogen environment generates the highest differences. Even if the absolute range of the relative fitness, *w*, is only in the order of 10^-3^, our competition experiments show that individuals with a better replacement size strategy are able to outcompete individuals with a slightly worse strategy (see also Additional file 1 Figure S2).

#### Replacement type

**Figure 6:**
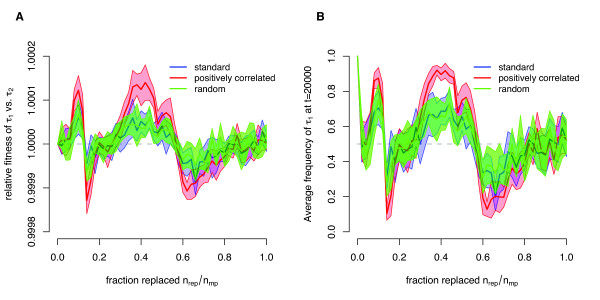
**Optimal replacement type**. **A **Relative fitness of replacement type *τ*_1 _= *age-dependent *against *τ*_2 _= *random *for the three different pathogen environments (*standard *(blue), *positively correlated *(red), *random *(green)) versus the fraction of memory cells replaced (*n*_rep_/*n*_mp_) with *n*_mp _= 50. The solid line denotes the average value for *w *over 15 simulations. The shaded area corresponds to the estimated pointwise 95%-confidence intervals. **B **Average frequency of individuals with replacement type *τ*_1 _in the total population at *t *= 20000.

To determine if the *age*-*dependent *replacement type provides a fitness advantage over the *random *replacement type, we also perform competition experiments with individuals differing only in this trait. We calculate the relative fitness advantage in 15 independent simulation runs considering replacement sizes from *n*_rep _= 1, 2,...,*n*_mp_. We also test the influence of the different pathogen environments on the results. Thereby we see that, for replacement sizes that cover approximately half of the total memory pool, there is a small relative fitness advantage for the *age*-*dependent *replacement type (see Figure 6). This effect is mostly observed for the positively correlated pathogen environment. However, there is no clear preference for the one or the other replacement type for specific fractions of memory pool replaced as the relative fitness differs very little from one. In general, the trend of the relative fitness estimated between different replacement types is independent of the pathogen environment. These findings are consistent for different memory pool sizes *n*_mp _(Additional file 1 Figure S3).

### Diversity

The optimal memory pool size, the optimal replacement size and the optimal replacement type also contain information about the diversity of the memory pool. An individual with a maximal memory pool size of *n*_mp _= 50 memory units, an optimal replacement size of *n*_rep _≈ 19 and the age-dependent replacement type can store memory cells specific for three to four different pathogens. As our optimal memory characteristics are robust with respect to the three different pathogen environments, this diversity estimate is independent of the correlations between occurrence and basic survival probability in the different pathogen environments. For the following section about diversity, we therefore constrain our analysis to the standard pathogen environment.

So far, we calibrated the pathogen environments such that individuals survive until the reproductive age with the probability *ζ *= 0.5. Because we set the infection probability *p*_inf _to 0.1, we expect the individuals to be infected on average with 4.5 pathogens until they begin to reproduce. The basic survival probability in the standard pathogen environment is therefore 0.86.

The number of pathogen encounters might play an important role for the development of immune memory. To study this effect, we analyze two additional scenarios for the standard pathogen environment. The probability to survive until the reproductive age, *ζ*, as well as the probability of a pathogen to infect an individual upon exposure, *p*_inf _, influence the number of encountered pathogens and may have an effect on the optimal replacement type *n*_rep _as well as on the diversity of the memory pool. To test this, we performed competition experiments with individuals possessing a maximal memory pool size of 50 memory units and using the age-dependent replacement type. In one scenario, we increase the probability to survive until the reproductive age, *ζ*, to 0.75 while the infection probability still is 0.1. This does not increase the number of pathogens encountered until the reproductive age, but more individuals survive until this age. The basic survival probability in this scenario is then 0.94. As a consequence, more individuals encounter a higher number of pathogens during their lifetime. In a second scenario, we test *ζ *= 0.75 and *p*_inf _= 0.5. The average number of pathogens encountered until the reproductive age is reached is 22.5. The basic survival probability in this scenario is then *p*_surv _= 0.99.

**Figure 7:**
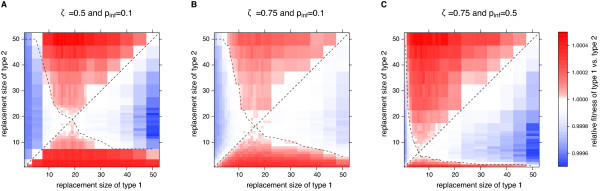
**Optimal replacement size under different parameterizations of the pathogen environment**. Simulations with age-dependent replacement type for the standard pathogen environment with different characteristics. **A **The parameters are the same as in our analysis above. The probability to survive until the reproductive age is *ζ *= 0.5 and the probability of infection *p*_inf _= 0.1. Hence, the calculated basic survival probability is *p*_surv _= 0.86. The optimal replacement size shifts to smaller values with smaller probability to survive until the reproductive age (**B**: *ζ *= 0.75, *p_inf _*= 0.1 and therefore *p*_surv _= 0.94) and with higher probability of infection (**C**: *ζ *= 0.75, *p_inf _*= 0.5 and therefore *p*_surv _= 0.99).

In Figure 7 we show the competition plots for (A) *ζ *= 0.5 and *p*_inf _= 0.1, (B) *ζ *= 0.75 and *p*_inf _= 0.1, (C) *ζ *= 0.5 and *p*_inf _= 0.1. The optimal replacement sizes are ≈19, ≈16 and ≈6 memory units, respectively. The diversity of the memory pool, i.e. the number of different pathogens against which the memory pool has stored memory cells, increases with decreasing replacement size and is 3 to 4, 4 to 5 and 9 to 10 for the different scenarios.

To explain the varying memory pool diversities in these different scenarios, we approximated the probability of an individual to become re-infected with the same pathogen and its expected lifetime. The respective formulae are derived in the Additional file 1 (see also Figure S1). The expected lifetimes in the different scenarios increase with increasing probability to survive until the reproductive age, *ζ*. The probability of re-infection during an individual's lifetime is the highest in scenario C. This means that the replacement size decreases with increasing probability to re-encounter a pathogen. In this case, it is better to evolve a more diverse memory pool, even at the cost of weaker protection against secondary infections.

## Discussion

An immune system is just one of the many evolutionary responses a host population may develop against the selection pressure by pathogens. If the pathogen environment consists of only a few, non-evolving pathogens the evolution of genetic resistance or tolerance might be a less costly alternative. An immune system, on the other hand, is a good evolutionary strategy against a very diverse or temporally variable pathogen environment. An adaptive immune system, which remembers previous pathogens and recalls specific responses, has the highest evolutionary benefit in a pathogen environment which changes from host generation to host generation, but is constant enough for multiple exposures with the same parasite strains to occur during the life-time of a host. The fact that vertebrates have evolved adaptive immune systems and arguably even invertebrates show evidence for acquired immune protection against secondary exposures [19,40,41] suggests that such pathogen environments are common. In this article, we investigated how memory of previous pathogen encounters can be stored optimally by individuals with adaptive immune systems. In particular, we investigated the optimal architecture of immune memory in the traits memory pool size, replacement size and replacement type. We developed an individual-based simulation model and performed in silico competition experiments between individuals with different immunological characteristics. Using these competition experiments, we studied the optimal memory traits for different pathogen environments.

To our knowledge, this study is the first to mathematically formalize pathogen environments that are more realistic in terms of their diversity and the stochastic patterns of exposure than previously conceived in theoretical studies. Interestingly, we found that the exact composition of the pathogen environments (parameterized by the occurrence probability and the basic survival probability) is less important for the evolution of different memory characteristics than we had anticipated. The average survival patterns (parameterized by the probability to survive until the reproductive age and the probability of infection) have a much stronger effect on the evolution of memory traits. These parameters strongly influence the probability to re-encounter a pathogen. The general conclusion of our modeling is the following: The more different pathogens individuals of a population are exposed to, the more diverse their memory pool should be, even if this increased diversity comes at the cost of poorer protection against the re-infection. To test this theoretical conclusion one could investigate to what extent host species differ in terms of their pathogen environments and if these differences could explain the observed variance in immune memory characteristics [42]. Specifically, our analysis suggests that populations inhabiting islands should be characterized by a less diverse immunological memory with stronger single responses than those that inhabit the mainland and are presumed to have larger and more diverse pathogen environments.

Arstilla et al. [43] studied the diversity of T cell receptors using combinatorial arguments. They estimated that the diversity of the T cell memory pool is about 1 × 10^5 ^to 2 × 10^5 ^epitope-specific T cell clones in humans. Given these estimates, the diversity of the memory pool in our simulations appears far too small. Partly this discrepancy is due to computational limitations, which make it difficult to perform simulations with thousands of pathogens in the environment of the hosts. We therefore chose to investigate the qualitative, rather than the quantitative relationship between the pathogen environment and the diversity in the memory compartment. We only studied the evolution of memory characteristics for pathogen environments with 50 different pathogens. Taylor et al. [44] and Woolhouse et al. [45] performed a systematic literature survey and found that 1399-1415 different pathogen species are known to be pathologic for humans and the number of pathogens with which humans are actually challenged will exceed the known number. However, it is important to realize that the memory units in our simulations are much more coarse-grained entities than T cell receptors. While one of our memory units remembers one pathogen, a T cell clone recognizes only one epitope of the pathogen, of which there are many. Thus, vertebrates remember fewer pathogens than their memory T cell diversity suggests. In order to relate the memory T cell diversity to the number of memorized pathogens, one would need an estimate of the number of epitopes that are recognized on pathogens on average.

Because the optimal replacement size is smaller than the maximal memory pool size, we observed an increase in the memory pool size with the first pathogen encounters. This is consistent with the findings in [28], namely that the memory compartment grows in size with immunological experience. However, we found that for the different pathogen environments infinite memory pool sizes are not required. Especially if there is a cost associated with immune memory (which is likely) a limited pool size will be favored from an evolutionary point of view. If the memory pool is limited, memory cells have to be replaced after the memory compartment has filled up. We conceived two replacement strategies: random and age-dependent replacement. These roughly correspond to the homeostasis model (in which the number of memory cells is reduced proportionally for each memory clone), and the attrition model (in which unrelated memory cells are reduced preferentially) [34,35], respectively. The consequences of a random replacement rule were already investigated in [37]. We do not find any evidence for selection in favor of any of these two replacement strategies. This may indicate that these strategies are evolutionarily neutral. However, due to the low numbers of cells in our memory pool, a potential selective advantage may be concealed by stochastic effects.

Taylor et al. [44] and Woolhouse et al. [45] studied risk factors and the ecological origins of human pathogens. However, the probabilities with which humans are challenged by the different pathogens (i.e. the "occurrence probability" in our model, or in epidemiological terms the "prevalence") and the probability to survive an infection with the pathogen (i.e. our "basic survival probability", or in epidemiological terms the "virulence") are unknown for most of the human pathogens. Therefore, our results have to remain qualitative and depend on our specific model assumptions.

Besides the characterization of real-life pathogen environments, there are several aspects concerning the generation of memory and the corresponding level of protection, which still have to be determined quantitatively. While for some acute infections, as e.g. measles and hepatitis A, recovery leads to lifelong immunity, in other diseases, as e.g. pertussis [46], immunity will only last for several years. For some vaccines, a sufficient protection is achieved after a single dose, while others need to be boosted [47]. The exact dose-response relationship between the amount of memory and the provided level of protection might vary between different pathogens and only a few studies investigated this relationship for specific pathogens [48]. In our simulations, we assumed that the beneficial effect of memory cells on the probability to survive an infection is concave downward (see Figure 2). This relation describes a scenario, in which even a small number of memory cells provides protection. Furthermore, we also assume that full protection against a pathogen is only reached if the total memory pool is specific for this pathogen. These two assumptions would correspond to a situation in which the successful survival of an acute infection provides a substantial benefit for the individual upon secondary exposure to the same pathogen. In addition, the longevity of memory in our model is determined by its replacement by other memory units, and can only be maintained by frequent re-stimulation. However, one could imagine different relationships as e.g. [49] showed that increased memory responses were associated with a decreased level of protection. Since this relationship between the amount of memory and level of protection is so central to the question of the evolution of immunological memory traits, it should be experimentally determined for different pathogens. One could adoptively transfer different numbers of memory cells against a given pathogen into mice and determine quantitatively how their survival against challenge with this pathogen is affected. Such an experiment would inform us if the protection is incremental, as we assume, or if a threshold has to be overcome.

Another important point, related to the quantity of memory responses and the level of protection, is the question of how many new memory cells are generated upon a secondary exposure to the same pathogen. In our model, we assume that the same amount of memory would be added to the memory pool as generated after the first exposure. The substantial growth of the memory compartment upon exposure has recently been shown experimentally [28,49]. However, one could also imagine a lower number of additional memory cells after re-exposure, dependent on the assumption of how memory cells are generated (reviewed in [50]). Many studies on HIV have shown that viral load and disease progression also depend on the genetic background of the host and not only on the pathogen strain transmitted [51,52]. Host heterogeneity might also play an important role in formation and maintenance of immune memory. In our model, all hosts with the same memory characteristics mount the same number of pathogens after the survival of an infection. Integrating host heterogeneity would complicate the models and introduce more variance into the results. Under the evolutionary pressure exerted on the hosts by the pathogen environment, first, immune-competent individuals will be selected, and among those, individuals with the optimal memory characteristics.

Our model also neglected complex processes of immunodynamics. We only considered acute infections by pathogens, which are cleared immediately and neglected persistence of pathogens. We also did not differentiate between different types of immune effectors or incorporated cross-reactivity or bystander effects. In the future, studies on the evolutionary ecology of immunological memory should incorporate more details on the dynamics of memory cells (as it was studied e.g. in [37,53,54]). In our model, memory cells are maintained until newly generated memory cells replace them. It would also be interesting to incorporate estimates of the life time of memory cells into our models [55], especially in order to understand differences between the duration of protection against different pathogens and why some vaccines need to be boosted [47].

In our study, the main selective force that affects immunological memory traits was the pathogen environment. However, other selective forces will also play a role. For example, the diversity of vertebrate immune system in general [56], and the specificity of immunological memory [57] is thought to be shaped by a trade-off between protection from pathogens and auto-immunity. Additionally, immune memory may be disadvantageous if it biases the secondary response to a closely related pathogen towards ineffective immune cell clones - a phenomenon called "original antigenic sin" [58]. Both of these factors will affect the diversity of the memory repertoire. In the future, these factors will have to be considered along with the primary selective pressure from the pathogen environment.

## Conclusions

In conclusion, we investigated the evolutionary optimality of immune memory traits under different pathogen environments with an individual-based model. Our results show, that the pathogen environment has a big influence on the optimal memory traits and as a consequence on the diversity of memory clones in the memory pool. The more frequently an individual is exposed to pathogens, the more diverse the memory pool should be, even if this diversity comes at the cost of reduced efficacy of immune memory.

## Authors' contributions

FG, CM and RRR designed the study and wrote the manuscript. FG and CM performed the simulations. All authors have read and approved the final manuscript.

## Supplementary Material

Additional file 1 **Relative Cost, ***ρ***, Re-infection Probability, Additional Figures**. In the Additional file 1 we describe how the relative cost, *ρ*, for different memory pool sizes is implemented in the model. We also show how the probability of being re-infected with the same pathogen and the expected lifetime of individuals can be approximated. Furthermore, three additional figures show how the relative fitness of individuals differing in their replacement type and the extinction probabilities are related, and how various parameters influence the optimal replacement types as well as the memory pool size without a cost. Click here for file
