# Correction: Theoretical analysis of the evolution of immune memory

**DOI:** 10.1186/1471-2148-11-54

**Published:** 2011-02-28

**Authors:** Frederik Graw, Carsten Magnus, Roland R Regoes

**Affiliations:** 1 Integrative Biology, ETH Zurich, Switzerland

## 

After the publication of this work [1], we became aware of the fact that the originally published Figure S-Three in the Additional file 1 was based on incorrectly simulated data. This simulation was accidentally performed with wrong parameter values for the total memory pool size *n*_mp_. As a consequence, Figure S-Three did not show the analysis we described in the figure legend. We repeated the simulations with the correct parameter values. The revised Figure S-Three does not change our conclusions and further corroborates the claims we made in the main manuscript (p.6 Results - Replacement type): "There is no clear preference for the one or the other replacement type for specific fractions of memory pool replaced as the relative fitness differs very little from one. In general, the trend of the relative fitness estimated between different replacement types is independent of the pathogen environment. These findings are consistent for different memory pool sizes *n*_mp_."

We regret any inconvenience that the incorrect Figure in the original article might have caused.

## Supplementary Material

Additional file 1 **Revised Figure S3 - Optimal replacement types**. Relative fitness *ω *of replacement type τ_1 _= *age-dependent against *τ_2 _= *random *for the three different pathogen environments (*standard *(blue), *positively correlated *(red), *random *(green)) given a total memory pool size of *n*_mp _= 25 **A **and *n*_mp _= 5 **C**, respectively. The solid line denotes the average value for *ω *over 15 simulations. The shaded area corresponds to the estimated pointwise 95%-confidence intervals. **B, D**. Average frequency of individuals with replacement type τ_1 _in the total population at *t *= 20000. Click here for file
