# Supplementary material for: Theoretical analysis of the evolution of immune memory
Source: BMC Evol Biol. 2010 Dec 8;10:380. doi: 10.1186/1471-2148-10-380 (PMC3018457; doi:10.1186/1471-2148-10-380)
Supplement: Additional file 1 — Relative Cost, ρ, Re-infection Probability, Additional Figures. In the Additional file 1 we describe how the relative cost, ρ, for different memory pool sizes is implemented in the model. We also show how the probability of being re-infected with the same pathogen and the expected lifetime of individuals can be approximated. Furthermore, three additional figures show how the relative fitness of individuals differing in their replacement type and the extinction probabilities are related, and how various parameters influence the optimal replacement types as well as the memory pool size without a cost. [file 1471-2148-10-380-S1.PDF]

Theoretical analysis of the evolution of immune memory Additional file 1

Frederik Graw[1,2], Carsten Magnus*[1,2] and Roland R Regoes[2]

[1] both authors contributed equally to this work
[2] Integrative Biology, ETH Zurich, Switzerland

Email: Frederik Graw - frederik.graw@env.ethz.ch; Carsten Magnus*- carsten.magnus@env.ethz.ch; Roland R Regoes - roland.regoes@env.ethz.ch;

*Corresponding author

The relative cost of different memory pool sizes

For the case that the generation of memory cells is associated with a cost, we assume that this cost affects the reproductive fitness. Individuals with a larger memory pool size, n_{mp}, have a lower reproduction probability λ. This relative cost, ρ denotes the ratio between the maximal reproduction probability $\lambda(n_{\mathrm{mp}} = 0)$ and the minimal reproduction probability $\lambda(n_{\mathrm{mp}} = 50)$, assuming a maximal memory pool size of $n_{\mathrm{mp}} = 50$. $\rho = 1$ describes a situation without costs, and $\rho = 2$ a situation in which an individual whithout memory has a reproduction probability λ that is twice as high as that of an individual with a maximal memory pool size $n_{\mathrm{mp}} = 50$. We assume that the loss in λ is linear in n_{mp}. With this definition, individuals having a maximal memory pool size of 50 memory units always have the same reproduction probability λ. For a fixed memory pool size smaller than 50, the probability to reproduce increases with increasing relative cost for generating memory, ρ.

Pathogen re-infection and expected lifetime of individuals

As seen in Figure 7 of the main text, the parameterization of our standard pathogen environment in terms of p_{inf} and ζ has an influence on the optimal replacement size $n_{\text{rep}}^{\text{optimal}}$ and thereby on the diversity of the individual memory pool. Based on the parameters defined in Table 1, the probability of re-infection with pathogen x, i.e. the probability that the pathogen survived in the first step, x, infects the individual for a second time at one of the following nth pathogen exposures is:

$$P(x(n)|x(0)) = \sum_{i=1}^{n} p_{\text{inf}} p_{\text{occ,x}} \left((1 - p_{\text{inf}}) + p_{\text{inf}}(1 - p_{\text{occ,x}}) p_{\text{surv}}\right)^{i-1} \tag{1}$$

Figure S1 shows the probability of re-infection for the different parameterizations of the standard pathogen environment as given in the legend of Figure 7. Comparing Figure S1 and $n_{\text{rep}}^{\text{optimal}}$ in Figure 7 we see, that the diversity of the individual memory pool is shaped by the re-infection probability as expected. For the first parameterization ($p_{\text{inf}} = 0.1, \zeta = 0.5$), we observe an optimal replacement size of $n_{\text{rep}}^{\text{optimal}} \approx 19$, which means that an individual can store memory against 3-4 different pathogens. An individual is re-infected with the same pathogen during its lifetime with a probability of 10%. However, the probability to encounter the same pathogen again when pathogen specific memory cells are still present and not replaced, is much smaller. The expected lifetime of an individual is given by

$$E[L] = \sum_{n=1}^{120} n * \nu(n) + 121 * \left(1 - \sum_{n=1}^{120} \nu(n)\right) \tag{2}$$

Hereby, $\nu(n)$ is the probability of an individual to die at the n-th pathogen exposure, given by

$$\nu(n) = \sum_{x=1}^{n} p_{\text{inf}}(1 - p_{\text{surv}}) \left((1 - p_{\text{inf}}) + p_{\text{inf}} p_{\text{surv}}\right)^{x-1} \tag{3}$$

The expected lifetime is usually smaller than the defined 40 years (=maximal age of reproduction). Hereby, the expected lifetime is calculated without considering the ability of an individual to gain additional protection by producing memory. For the different parameterizations of the standard pathogen environment, we calculated expected lifetimes for individuals of 19.25 years ($p_{\text{inf}} = 0.1, \zeta = 0.5, p_{\text{surv}} = 0.86$), 28.44 years ($p_{\text{inf}} = 0.1, \zeta = 0.75, p_{\text{surv}} = 0.94$) and 28.20 years ($p_{\text{inf}} = 0.5, \zeta = 0.75, p_{\text{surv}} = 0.99$).

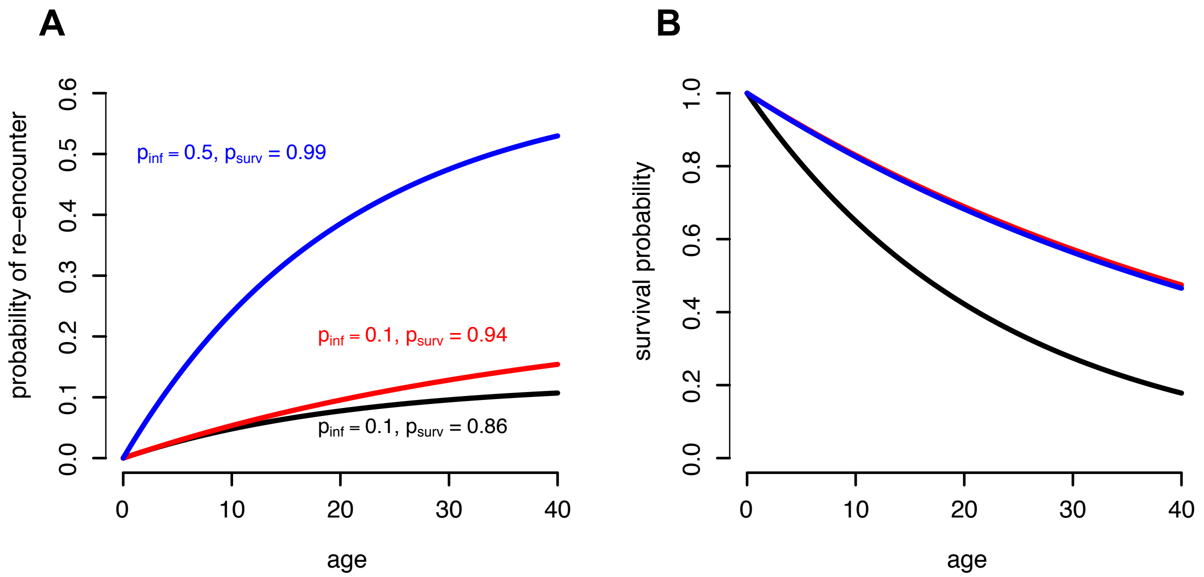

Figure S1 - Re-infection probability

Probability to become re-infected with a pathogen after n subsequent pathogen exposures for different parameterizations of the standard pathogen environment. **B** Corresponding survival probability of an individual without considering the ability to develop memory.

Additional Figures

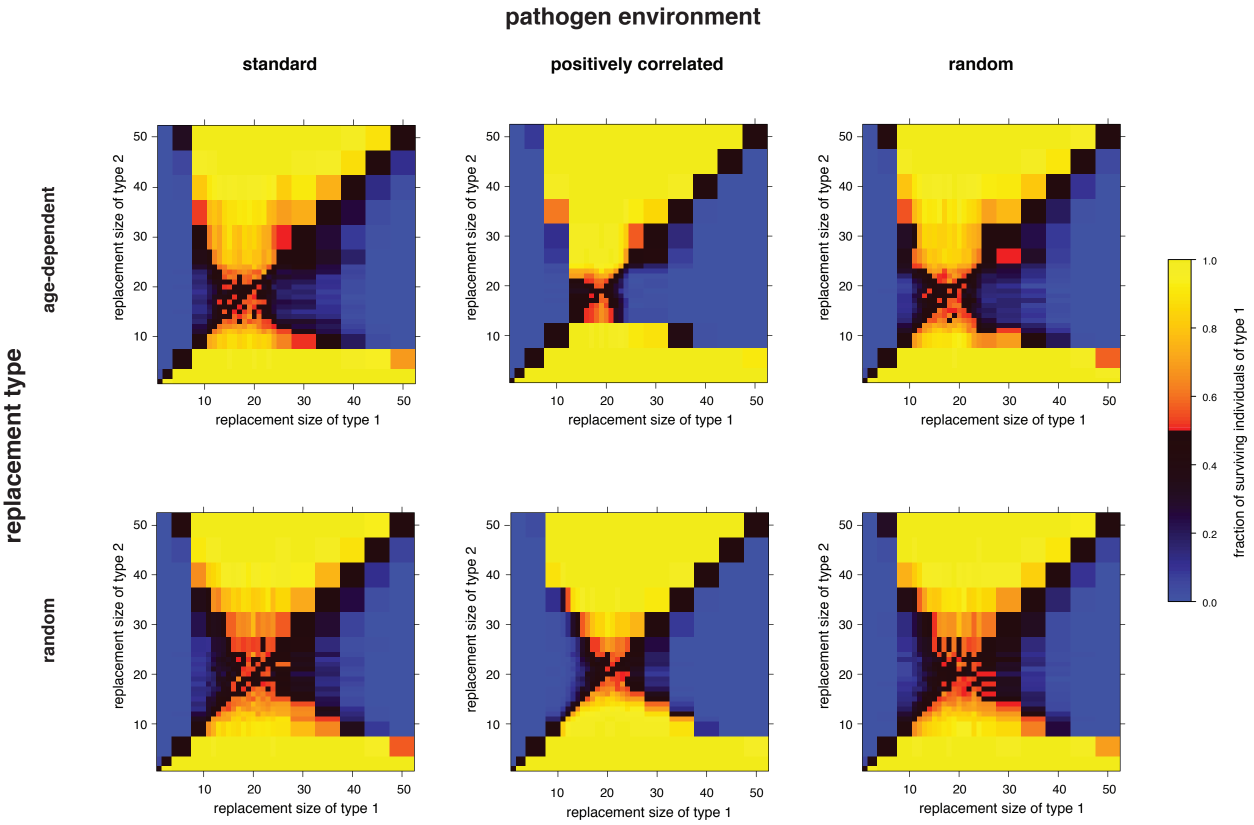

Figure S2 - Pairwise extinction

Pairwise extinction plots for the three pathogen environments and the two replacement types. For each replacement size combination, the fraction of individuals of type 1 after 20000 time steps averaged over 15 independent simulations is depicted. For yellowish colors, the fraction of individuals of type 1 is higher and for blueish colors, the fraction of individuals with replacement size 2 is higher. Even small differences in fitness (see Figure 5) result in the eventual extinction of one individual type with a high probability.

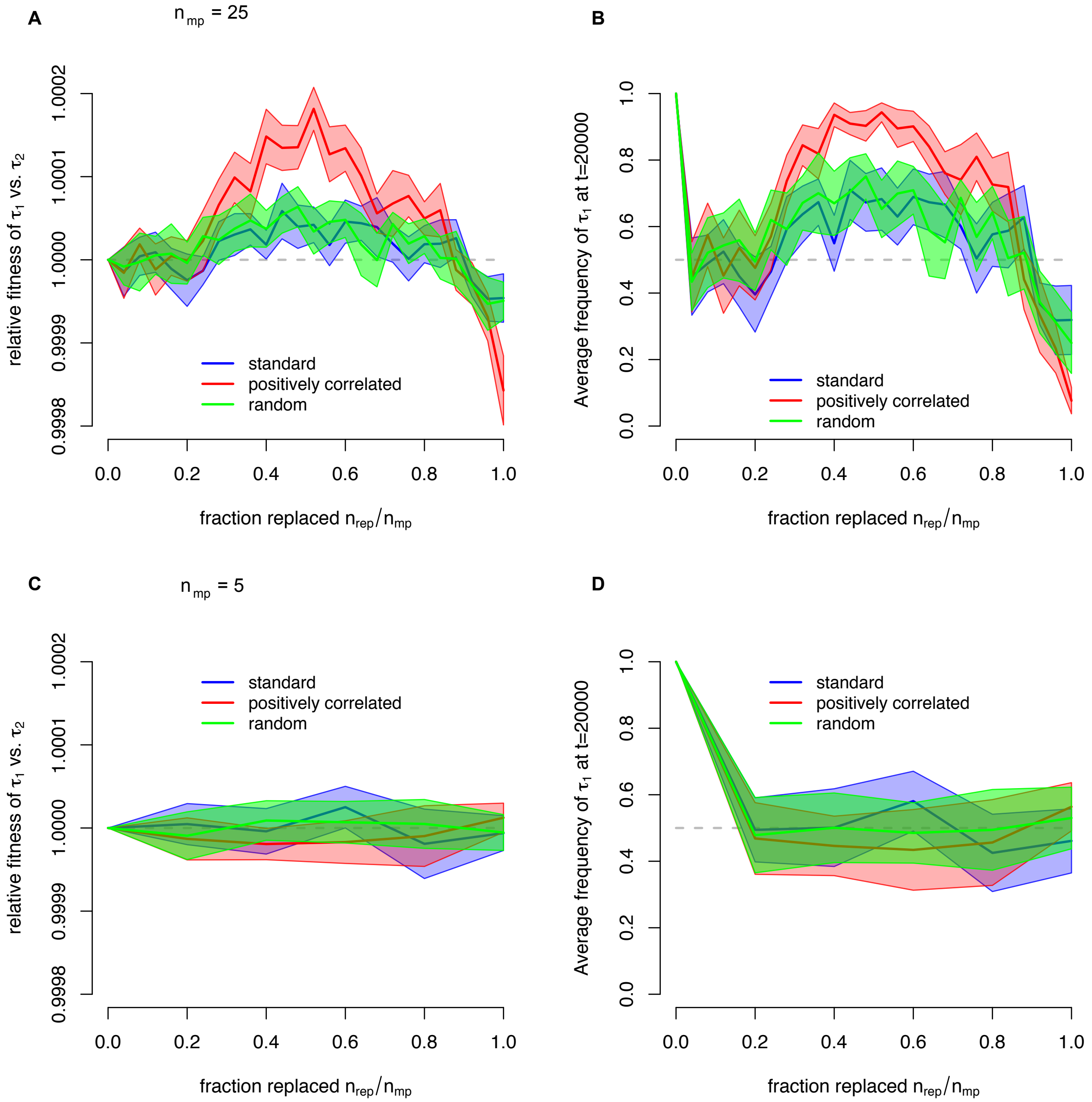

Figure S3 - Optimal replacement types

Relative fitness w of replacement type τ_1 =*age-dependent* against τ_2 =*random* for the three different pathogen environments (*standard* (blue), *positively correlated* (red), *random* (green)) given a total memory pool size of $n_{\mathrm{mp}} = 25$ **A** and $n_{\mathrm{mp}} = 5$ **C**, respectively. The solid line denotes the average value for w over 15 simulations. The shaded area corresponds to the estimated pointwise 95%-confidence intervals. **B, D** Average frequency of individuals with replacement type τ_1 in the total population at $t = 20000$.

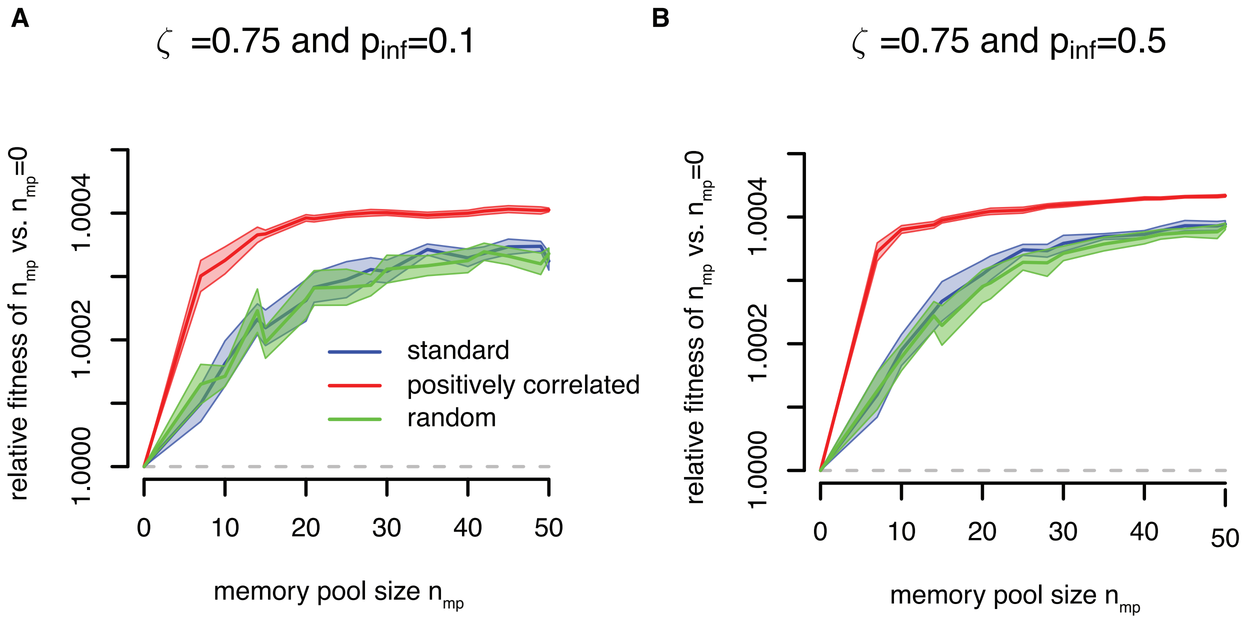

Figure S4 - Evolution of memory pool size without a cost

Effect of memory pool size without a cost: Relative fitness as a function of the memory pool size n_{mp}. The relative fitness of individuals with a memory pool size $n_{\mathrm{mp}} = 0$ is normalized to 1. The mean (solid line) as well as the 95% confidence intervals (shaded area) over 15 simulation runs are shown for each pathogen environment. The replacement size was fixed to $n_{\mathrm{rep}} = 7$. The survival probability until the reproductive age ζ and the probability of infection upon exposure vary in comparison to the parameters of Figure 3 in the main text: **A** $\zeta = 0.75$, $p_{\mathrm{inf}} = 0.1$ and **B** $\zeta = 0.75$, $p_{\mathrm{inf}} = 0.5$
