# Supplementary figures and images for: Correction: Theoretical analysis of the evolution of immune memory
Source: BMC Evol Biol. 2011 Feb 28;11:54. doi: 10.1186/1471-2148-11-54 (PMC3050725; doi:10.1186/1471-2148-11-54)

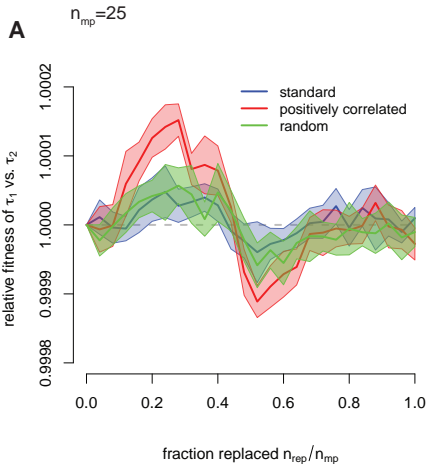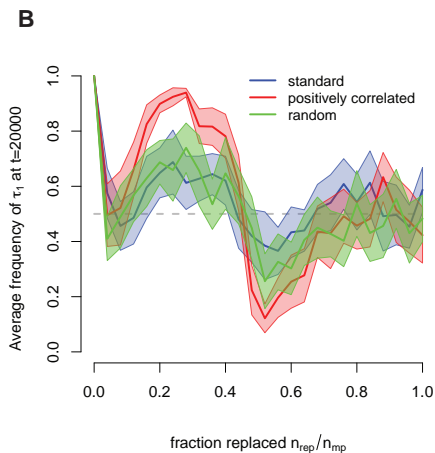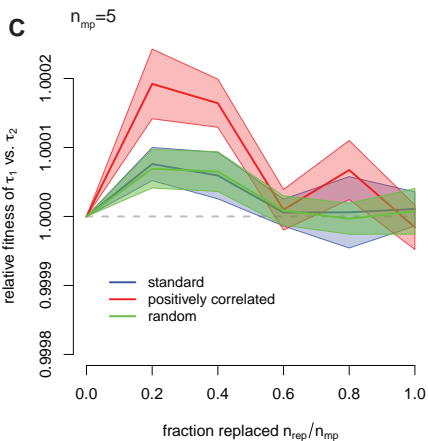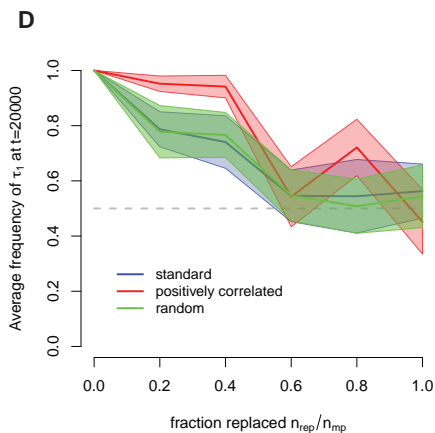

Supplement: Additional file 1 — Revised Figure S3 - Optimal replacement types. Relative fitness ω of replacement type τ1 = age-dependent against τ2 = random for the three different pathogen environments (standard (blue), positively correlated (red), random (green)) given a total memory pool size of nmp = 25 A and nmp = 5 C, respectively. The solid line denotes the average value for ω over 15 simulations. The shaded area corresponds to the estimated pointwise 95%-confidence intervals. B, D. Average frequency of individuals with replacement type τ1 in the total population at t = 20000. [file 1471-2148-11-54-S1.PDF]
